# Alcohol Consumption and Longitudinal Trajectories of Physical Functioning in Central and Eastern Europe: A 10-Year Follow-up of HAPIEE Study

**DOI:** 10.1093/gerona/glv233

**Published:** 2016-01-08

**Authors:** Yaoyue Hu, Hynek Pikhart, Ruzena Kubinova, Sofia Malyutina, Andrzej Pajak, Agnieszka Besala, Steven Bell, Anne Peasey, Michael Marmot, Martin Bobak

**Affiliations:** ^1^Research Department of Epidemiology and Public Health, University College London.; ^2^National Institute of Public Health, Prague, Czech Republic.; ^3^Institute of Internal and Preventive Medicine, Novosibirsk, Russia.; ^4^Novosibirsk State Medical University, Russia.; ^5^Department of Epidemiology and Population Studies, Faculty of Health Sciences, Jagiellonian University Medical College, Krakow, Poland.

**Keywords:** Alcohol consumption, Physical functioning trajectories, Central and Eastern Europe

## Abstract

**Background:**

Physical functioning (PF) is an essential domain of older persons’ health and quality of life. Health behaviors are the main modifiable determinants of PF. Cross-sectionally, alcohol consumption appears to be linked to better PF, but longitudinal evidence is mixed and very little is known about alcohol consumption and longitudinal PF trajectories.

**Methods:**

We conducted longitudinal analyses of 28,783 men and women aged 45–69 years from Novosibirsk (Russia), Krakow (Poland), and seven towns of the Czech Republic. At baseline, alcohol consumption was measured by a graduated frequency questionnaire and problem drinking was evaluated using the CAGE questionnaire. PF was assessed using the Physical Functioning Subscale of the SF-36 instrument at baseline and three subsequent occasions. Growth curve modeling was used to estimate the associations between alcohol consumption and PF trajectories over 10-year follow-up.

**Results:**

PF scores declined during follow-up in all three cohorts. Faster decline in PF over time was found in Russian female frequent drinkers, Polish female moderate drinkers, and Polish male regular heavy drinkers, in comparison with regular and/or light-to-moderate drinkers. Nondrinking was associated with a faster decline compared with light drinking only in Russian men. Problem drinking and past drinking were not related to the decline rate of PF.

**Conclusions:**

This large longitudinal study in Central and Eastern European populations with relatively high alcohol intake does not strongly support the existence of a protective effect of alcohol on PF trajectories; if anything, it suggests that alcohol consumption is associated with greater deterioration in PF over time.

Physical functioning (PF) is a key indicator of older persons’ health status and is strongly related to their quality of life. Decline in PF is a consequence of age-dependent physiological changes and onset of diseases and can be modified by medical care, socioeconomic, environmental, psychosocial, and behavioral factors (1). Changes in body composition with increasing age (increased body fat and decreased body water) and negative interactions between alcohol and medications can elevate older persons’ susceptibility to harmful impacts of alcohol (2).

Cross-sectional studies have shown a protective effect of light-to-moderate drinking on PF (3–5), yet there are concerns about reverse causation and other biases (6–8). Some prospective studies have accorded with the cross-sectional findings reporting lower PF or a heightened risk of functional limitations and physical disability in non and/or heavy drinkers than in light-to-moderate drinkers (9,10); whereas other prospective studies have found no association (11,12). Moreover, published studies have usually only examined alcohol consumption in relation to functional limitations/disability at one or two time points, rather than PF trajectories over time. Abstainers and heavy drinkers, compared with light-to-moderate drinkers, are more likely to have lower socioeconomic status (SES) (7), and lower SES has been shown to be a risk factor of PF in older populations (13). Previous studies have controlled for the possible confounding effect of SES, but SES may potentially also modify the association between alcohol consumption and PF. Most of these studies were from western countries, and none used Central and Eastern European (CEE) populations.

The rapid population aging in CEE brings major public health challenges, given the often inadequate provision of health services and long-term social care and low private savings (14). Compared with Western Europe, CEE has a shorter life expectancy (15), higher alcohol consumption (16), and a larger alcohol-attributable burden of ill health (17). Older persons’ PF in CEE also appears to be poorer than their western counterparts (18), which may be attributable to their high alcohol intake.

In this study, we investigated individual PF trajectories in three aging cohorts in CEE over approximately 10 years and how the trajectories were influenced by alcohol consumption, problem drinking and past drinking behavior.

## Methods

### Study Participants

We used data from the Health, Alcohol and Psychosocial factors In Eastern Europe (HAPIEE) study in Novosibirsk (Russia), Krakow (Poland), and seven middle-sized towns in the Czech Republic (Havířov/Karviná, Jihlava, Ústí nad Labem, Liberec, Hradec Králové, and Kromĕříz) (19). In 2002–2005 (baseline), 28,783 men and women aged 45–69 years were randomly selected from population registers in Czech towns and Krakow and electoral lists in Novosibirsk, stratified by sex and 5-year age bands, with an overall response rate of 60% (19). Czech and Polish participants completed a structured questionnaire at home and then were invited to a short medical examination in a clinic. Russian participants completed both with nurses during their visits to a clinic. The questionnaire was translated into local languages and back-translated into English to ensure accuracy and cross-cultural comparability. Participants were re-examined in 2006–2008 by face-to-face Computer Assisted Personal Interview and followed-up by postal questionnaires in 2009 (PQ2009) and 2012 (PQ2012), respectively. The HAPIEE study was approved by ethics committees at University College London and all local centers. All participants gave their written informed consent.

### Physical Functioning

PF was measured at all four occasions using the Physical Functioning Subscale (PF-10) of the Short-Form-36 instrument (20). The PF-10 assesses limitations on 10 items regarding vigorous and moderate activities, lifting groceries, mobility, and self-care tasks. Participants rated themselves as “limited a lot,” “limited a little,” or “not limited at all” to each item. A summated score (0–100) was derived, with higher score indicating better PF (21).

### Alcohol Consumption

Alcohol consumption in the 12 months before baseline was assessed using a graduated frequency (GF) questionnaire (22). Six drinking quantities during 1 day (≥10, 7–9, 5–6, 3–4, 1–2, and 0.5 drink) were asked, with nine drinking frequencies provided for each quantity (every day or almost every day, 3–4/week, 1–2/week, 2–3/month, 1/month, 6–11/year, 3–5/year, 1–2/year, and never in the past year). One standard drink was defined as 0.5L of beer, 2 dL of wine, or 5 cL of spirits, each containing about 20g of alcohol.

Average drinking frequency, annual drinking volume, and average drinking quantity per drinking day were calculated from the GF using midpoints of drinking quantities and corresponding drinking frequencies. Average drinking quantity per drinking day was categorized into non, light, moderate, and heavy drinking (0, 0.1–19.9, 20.0–39.9, ≥40.0g/day for women; 0, 0.1–39.9, 40.0–59.9, ≥60.0g/day for men (23)). Drinking pattern was obtained directly from the GF. Light-to-moderate drinking was defined as ≤4 drinks/day (≤2 drinks/day for women); higher intakes were categorized as heavy drinking. Regular drinking was defined as ≥1/week; less than this was coded as irregular drinking. In women, regular versus irregular heavy drinkers were classified using 1/month, as very few female heavy drinkers drank ≥1/week. Problem drinking at baseline was evaluated using the CAGE (24) and identified by having ≥2 positive responses (25).

Among Russians, past drinking was determined by a question asking whether participants had cut down their drinking frequencies before baseline. Nondrinkers identified by the GF were then separated into former drinkers and lifetime abstainers. Current drinkers were split into those who had reduced drinking and those who had maintained drinking. According to self-reported reasons for reducing drinking, former drinkers and reduced drinkers were further categorized into due to health versus nonhealth reasons.

### Covariates

Several baseline characteristics were included as covariates. Age was classified into 5-year groups. Marital status was dichotomized into married/cohabiting versus others. Educational attainment (university, secondary school, and <secondary school), sum of ownership of 12 household amenities (eg, mobile phone and washing machine), and economic activity were selected to reflect participants’ SES. Economic activity included four categories: working, pensioners but still employed, pensioners without employment, and unemployed. Spine/joint problems were based on self-reported diagnosis/hospitalization for a disease of spine or joints in the past year. Objectively measured height (m) and weight (kg) were used to calculate body mass index. Smoking status was coded as never, former, and current smoking.

### Statistical Analysis

Missing data were mainly on the PF-10 scores during follow-up (Supplementary Table 1). Missing data were imputed using multiple imputation by chained equations, and 70 imputed data sets were generated in Stata 12 (StataCorp, 2013) (26). Most participants (76%) provided their PF for at least two measurement occasions, with high correlations between occasions (correlation coefficients: 0.53–0.73). PF-10 scores from other occasions were included in the imputation models; this further improves the reliability of the imputed scores. Missing follow-up years due to nonresponse to follow-up were substituted by random numbers generated under normal distributions of observed follow-up years.

Intra-individual PF-10 changes (trajectories) over the 10-year follow-up and interindividual variations in the trajectories were estimated using growth curve modeling (27). The shape of the trajectories was determined by comparing linear and quadratic models. In Czech men and Russians, the quadratic models fitted the data statistically better than linear models. Given the large sample sizes, even relative small and clinically unimportant differences between models are likely to reach statistical significance. We found that the 10-year PF declines were very similar in both models. Considering that linear models are easier to interpret, linear trajectories are presented. The multiply imputed data sets were analyzed using M*plus* 6.0 (Muthén & Muthén, 1998–2011). Pooled estimates based on these imputed data sets are presented. Two growth parameters describe the trajectories: initial status (PF-10 score at baseline) and slope (rate of change in PF-10 score per year of follow-up). Maximum likelihood estimation with robust standard errors was used due to the non-normality of the PF-10 scores. Two models were fitted by cohort and sex separately for each drinking index, adjusting for (i) age (Model 1) and (ii) age, marital status, education, household amenities, economic activity, spine/joint problems, body mass index, and smoking status (Model 2). In additional models, we also examined the interaction between alcohol consumption indices and socioeconomic factors.

## Results

Table 1 summarizes the average sample characteristics in the 70 imputed data sets; the observed characteristics and proportions of missing data at each measurement occasion are shown in Supplementary Table 1. Baseline PF-10 scores were higher among men than among women and declined during follow-up in both sexes. More men were drinkers than women, and they drank more frequently and heavily. Very few women were identified as problem drinkers; thus, the relationship between problem drinking and PF-10 trajectories was not examined among women.

**Table 1.** Physical Functioning and Alcohol Consumption Characteristics in the Imputed Data Sets

	Country					
	Czech Republic		Russia		Poland	
	Men	Women	Men	Women	Men	Women
Total	4,070	4,703	4,239	5,062	5,219	5,490
PF-10 score (mean, *SD*)						
Baseline	85.1 (18.2)	81.8 (19.4)	86.9 (18.3)	77.5 (21.1)	83.9 (20.2)	77.0 (21.9)
Re-examination	83.5 (17.2)	80.5 (18.8)	84.7 (20.6)	75.5 (22.9)	76.8 (20.4)	71.2 (21.8)
PQ2009	80.0 (22.9)	77.5 (23.0)	75.2 (26.8)	63.4 (27.1)	75.3 (26.0)	66.6 (27.2)
PQ2012	78.3 (24.0)	76.5 (24.3)	69.2 (29.6)	59.5 (29.0)	70.0 (26.6)	61.6 (27.7)
Average drinking quantity/drinking day (%)						
Nondrinker	6.5	18.6	13.5	17.8	22.0	46.4
Light	66.5	33.7	24.0	19.0	58.6	29.9
Moderate	9.5	37.9	18.2	49.4	7.0	20.3
Heavy	17.4	9.8	44.4	13.8	12.5	3.5
Drinking pattern (%)						
Nondrinker	6.6	18.6	13.5	17.8	21.9	46.3
Irregular light-to-moderate	22.9	39.5	23.8	58.7	27.7	35.2
Regular light-to-moderate	27.9	12.5	17.5	4.3	22.6	7.3
Irregular heavy	34.9	18.8	31.3	13.0	24.3	7.7
Regular heavy	7.8	10.5	13.9	6.2	3.5	3.4
Problem drinking (%)						
No	90.8	98.0	80.8	98.6	91.0	99.0
Yes	9.2	2.0	19.2	1.4	9.0	1.0

*Note:* PF-10 = Physical Functioning Subscale; SD = standard deviation.

Table 2 presents the multivariable-adjusted associations of alcohol consumption with PF-10 trajectories. The PF-10 scores at baseline (initial status, corresponding to cross-sectional associations) were consistently the lowest among nondrinkers, and they increased with increasing level of alcohol consumption. Among male drinkers, problem drinking was related to a higher score at baseline in Russian men. In the Russian cohort, the lowest PF-10 score at baseline was observed in former drinkers who quit drinking for health reasons.

**Table 2.** Multivariable-adjusted Associations Between Alcohol Consumption and Physical Functioning Trajectories

	Men (coefficient, *SE*)			Women (coefficient, *SE*)		
	Czech Republic	Russia	Poland	Czech Republic	Russia	Poland
Initial status^a^	93.38 (1.22)***	91.92 (1.37)***	89.99 (1.31)***	89.95 (1.35)***	90.46 (1.66)***	93.05 (1.59)***
Slope^a^	−0.21 (0.22)	−0.62 (0.33)	−1.26 (0.27)***	−0.18 (0.23)	−1.84 (0.35)***	−1.58 (0.31)***
Average drinking quantity/drinking day (reference group: light drinker)						
Initial status						
Nondrinker	−6.05 (1.38)***	−1.74 (1.02)	−3.42 (0.72)***	−4.10 (0.80)***	−5.55 (0.98)**	−2.64 (0.63)**
Moderate	0.98 (0.74)	3.05 (0.73)***	2.00 (0.81)*	0.36 (0.50)	1.56 (0.69)*	2.22 (0.64)**
Heavy	0.07 (0.64)	3.10 (0.65)***	1.68 (0.71)*	0.08 (0.76)	2.54 (0.91)**	2.37 (1.11)*
Slope						
Nondrinker	−0.03 (0.21)	−0.41 (0.21)*	−0.02 (0.13)	−0.08 (0.13)	−0.02 (0.18)	−0.10 (0.12)
Moderate	−0.26 (0.14)	−0.26 (0.16)	−0.19 (0.17)	0.01 (0.08)	−0.10 (0.14)	−0.24 (0.12)*
Heavy	−0.04 (0.11)	−0.24 (0.14)	−0.18 (0.14)	0.00 (0.13)	−0.03 (0.18)	−0.28 (0.23)
Drinking pattern (reference group: regular light-to-moderate drinker)						
Initial status						
Nondrinker	−6.17 (1.42)***	−4.17 (1.01)***	−3.30 (0.81)***	−4.52 (0.95)***	−8.52 (1.35)***	−5.32 (0.97)***
Irregular light-to-moderate	−1.11 (0.68)	−2.59 (0.78)**	−0.32 (0.65)	−0.89 (0.71)	−2.16 (1.13)	−2.20 (0.92)*
Irregular heavy	0.85 (0.58)	1.14 (0.67)	2.29 (0.62)***	1.14 (0.76)	0.45 (1.25)	−1.71 (1.11)
Regular heavy	−0.14 (0.94)	0.69 (0.80)	1.06 (1.30)	0.10 (0.84)	−1.54 (1.44)	0.50 (1.41)
Slope						
Nondrinker	−0.10 (0.21)	−0.25 (0.21)	−0.08 (0.15)	−0.19 (0.15)	0.40 (0.27)	0.08 (0.18)
Irregular light-to-moderate	−0.16 (0.12)	0.08 (0.18)	−0.01 (0.12)	−0.11 (0.11)	0.38 (0.23)	0.13 (0.17)
Irregular heavy	−0.11 (0.10)	−0.01 (0.17)	−0.24 (0.14)	−0.11 (0.13)	0.43 (0.26)	−0.06 (0.22)
Regular heavy	−0.29 (0.18)	−0.17 (0.21)	−0.63 (0.30)*	−0.12 (0.15)	0.17 (0.30)	−0.13 (0.28)
Problem drinking^b^ (reference group: nonproblem drinking)						
Initial status						
Problem drinking	−0.68 (0.85)	1.31 (0.60)*	−0.86 (0.88)			
Slope						
Problem drinking	−0.09 (0.14)	−0.11 (0.16)	−0.20 (0.20)			
Past drinking behavior (reference group: continuing drinker)						
Intercept						
Lifetime abstainer		−4.68 (2.91)			−4.77 (1.11)***	
Former drinker, health reasons		−11.42 (1.59)***			−12.70 (1.49)***	
Former drinker, nonhealth reasons		−0.62 (0.93)			−6.23 (1.51)***	
Reduced drinker, health reasons		−7.72 (0.84)***			−5.53 (0.94)***	
Reduced drinker, nonhealth reasons		0.30 (0.56)			1.04 (0.69)	
Slope						
Lifetime abstainer		0.10 (0.55)			0.02 (0.21)	
Former drinker, health reasons		−0.01 (0.29)			0.10 (0.25)	
Former drinker, nonhealth reasons		−0.36 (0.22)			0.25 (0.27)	
Reduced drinker, health reasons		0.08 (0.19)			0.29 (0.17)	
Reduced drinker, nonhealth reasons		0.15 (0.13)			0.14 (0.14)	

*Notes:* Adjusted for age, educational attainment, household amenities, economic activity, marital status, spine/joint problems, body mass index, and smoking status.

SE: standard error.

^a^Conditional on covariates.

^b^Among male drinkers.

*p < .05. **p < .01. ***p < .001.

Czech participants’ PF-10 scores declined more slowly than among their Russian and Polish counterparts (slope, corresponding to longitudinal trajectories) (*p* < .001). Compared with the age-adjusted models (Supplementary Table 2), some associations between alcohol consumption and the slope became statistically insignificant after multivariable adjustment. In most country-sex groups, the slope did not significantly differ across drinking categories. The PF-10 scores in Polish male regular heavy drinkers declined more steeply than in regular light-to-moderate drinkers (difference in the slope: −0.63, *SE* = 0.30). Polish female moderate drinkers were also found experiencing a faster decline than light drinkers (−0.24, *SE* = 0.12). In Russian men, compared with light drinking (average drinking quantity/drinking day), nondrinking was related to a faster decline (−0.41, *SE* = 0.21), but this was not replicated in other drinking indices. Multivariable-adjusted results on drinking frequency and annual drinking volume are provided in Supplementary Table 3. Among Russian women, a more rapid PF decline was observed in those who drank ≥1/week versus 1–3/month (−0.37, *SE* = 0.19). We found no significant associations of the slope of PF decline with problem drinking among male drinkers or with past drinking behavior among Russians. This pattern was replicated after further categorizing past drinking by drinking pattern (Supplementary Table 4).

Further adjustment for baseline health conditions reduced the differences in the baseline PF-10 scores between drinking categories, but the associations of alcohol with the slope changed very little (Supplementary Table 5). There were no statistically significant interactions between SES (education and ownership of household amenities) and alcohol consumption on the slope of PF decline (not shown in table).

## Discussion

In this study, we assessed associations between longitudinal PF trajectories and alcohol consumption in three population-based cohorts in CEE. We found a slower PF decline in Czechs than in Russians and Poles. Decline rates were not systematically associated with alcohol intake, problem drinking or past drinking, although in some, but not all subgroups, the PF decline appeared somewhat faster among frequent and heavier drinkers than in light-to-moderate drinkers.

Several limitations in this study need to be acknowledged. First, measurement of alcohol intake in epidemiological studies is subject to measurement error (6,28). The GF requires respondents to remember their drinking occasions correctly in the past year and distribute them accurately over drinking quantities (28). Participants may not be able to recall their alcohol consumption precisely and underreport their consumption (28). This underreporting may increase with increasing level of alcohol intake (29). Moreover, participants with insufficient cognitive skills may be less likely to respond to the GF correctly (30). Although self-reported alcohol consumption generally only covers 30%–70% of sales data on alcohol (28), it is reasonably reliable to rank people by their “true” consumption (28). The reliability of the GF and CAGE in this study was supported by the positive associations of GF-based indices and problem drinking with serum gamma-glutamyl transferase (Supplementary Table 6).

Second, participants might overreport their PF, particularly when being interviewed, because of the stigma attached to being unhealthy (31). In earlier reports, PF-10 scores were lower when the SF-36 was administrated by post than in interviews (32,33). The PF decline in our study thus may be overestimated due to the change of assessment mode. To account for the different measurement errors, we constrained the residuals of PF-10 scores at baseline and re-examination to be equal in the growth curve models, and similarly for the residuals at PQ2009 and PQ2012. This potential overestimation, however, is unlikely to bias the association with alcohol, as the assessment mode was identical for all participants, irrespective of alcohol consumption. Moreover, the fact that the PF-10 score was strongly associated with the objective assessments of grip strength and chair rise at re-examination supports its validity (Supplementary Table 7).

The third methodological concern is residual confounding. In western societies, both abstainers and heavy drinkers tend to have lower SES, poorer health, poorer social network, and less favorable behavior than moderate drinkers, and it is difficult to entirely control for all these factors (7). In our data, we found little evidence of confounding by baseline health conditions, and there was no evidence that the effect of alcohol on PF decline was modified by SES. Given the weak and inconsistent associations of drinking indices with PF trajectories in this data, it is unlikely that the inclusion of incident events during follow-up in the model would change the results.

Finally, middle-class individuals with relatively favorable drinking patterns and health profile are likely to be overrepresented in studies (34); whereas heavy drinkers are likely to be underrepresented (29). The possible underrepresentation of heavy drinkers may partly explain the lack of consistent findings in this study.

To our knowledge, this is one of the first investigations of the relationship between alcohol consumption and PF trajectories in aging populations. The multicenter design optimizes the cross-country comparability in this study. Unlike most existing studies, we examined several aspects of alcohol consumption, including drinking patterns, which may have a major influence on health in CEE, particularly in Russia (35). Data on past drinking, although available only in Russians, are valuable for assessing the influence of past drinking and reasons for reduction/cessation on PF. Such data have not been available (or used) in the majority of previous studies of alcohol consumption and PF.

Our findings are generally consistent with a recent study that found no associations of alcohol consumption with either the onset or rate of change in the severity of functional limitations in middle-aged Americans over 10 years (12). By contrast, Wang and colleagues (9) reported a slower PF decline over a 4-year period among older adults who drank ≥5 drinks in the past year compared with those who consumed <5 drinks. However, the crude measurement of alcohol consumption in Wang and colleagues’ study (whether consumed ≥5 drinks in the past year) may introduce considerable misclassification error. Besides, the inconsistency between studies may also be due to differences in age structure between cohorts.

The discrepancies in PF decline between the three cohorts may reflect a better population health in the Czech Republic than in Russia and Poland. This is exemplified by the highest life expectancy reported in the Czech Republic, followed by Poland, and lastly Russia (15). In addition, Czechs are frequent drinkers, mainly beer drinkers; by contrast, Russians (particularly men) are mostly vodka drinkers with episodic consumption of large quantities (16,36). These differences in drinking culture may be linked with differential measurement error of alcohol consumption and thus differentially bias the decline rates in PF across drinking categories between cohorts. Given the high prevalence of infrequent drinking in Russian women (46%), those who drink frequently may be a particularly select subgroup with high alcohol intake and/or poor health. This may partly explain the faster PF decline found in frequent female drinkers in Russia. Finally, PF and its reporting can be influenced by medication, rehabilitation, and accommodation (eg, use of assistive device and removal of obstacles/barriers) (37). Differential access to such interventions may also explain differences in PF trajectories between cohorts and over time.

An important issue to consider is the potential bidirectionality of the association between alcohol and PF. Heavy drinkers at baseline might have been able to maintain high consumption because of their good health. During follow-up, once their health deteriorated, they might reduce or stop drinking, and the process of PF decline may slow down or even reverse. This bidirectional relationship may contribute to heavy drinkers’ good PF at baseline but no consistently faster decline during follow-up. We could not explore such bidirectionality because alcohol consumption was not measured repeatedly in this study. Future studies with repeated measurements of alcohol consumption therefore are needed.

## Conclusions

PF declined during 10-year follow-up in all three aging cohorts, but alcohol consumption was not consistently associated with the decline rate. These longitudinal analyses did not provide strong support for the existence of protective effect of alcohol shown in cross-sectional studies; if anything, they suggested that alcohol consumption was associated with faster PF decline over time. Considering the detrimental effect of alcohol consumption on numerous diseases (38), it seems sensible to encourage older adults to reduce/stop drinking alcohol. Future research with repeated measurements on both alcohol consumption and PF is needed to clarify whether the absence of association between heavy drinking and faster PF decline is genuine or whether detection of such an association requires better measurement and longer follow-up.

## Supplementary Material

Please visit the article online at http://gerontologist.oxfordjournals.org/ to view supplementary material.

## Funding

The HAPIEE study was supported by Wellcome Trust “Determinants of Cardiovascular Diseases in Eastern Europe: Longitudinal follow-up of a multi-centre cohort study” (The HAPIEE Project) (Reference number 081081/Z/06/Z); MacArthur Foundation “Health and Social Upheaval (a research network)”; and the U.S. National Institute on Aging “Health disparities and aging in societies in transition (the HAPIEE study)” (Grant number 1R01 AG23522). S.B. is supported by the European Research Council (ERC-StG-2012-309337) and UK Medical Research Council/Alcohol Research UK (MR/M006638/1).

## Supplementary Material

Supplementary Data
