# Supplementary material for: Alcohol Consumption and Longitudinal Trajectories of Physical Functioning in Central and Eastern Europe: A 10-Year Follow-up of HAPIEE Study
Source: J Gerontol A Biol Sci Med Sci. 2016 Jan 8;71(8):1063–8. doi: 10.1093/gerona/glv233 (PMC4945885; doi:10.1093/gerona/glv233)
Supplement: Supplementary Data [file supp_71_8_1063__index.html]

Alcohol Consumption and Longitudinal Trajectories of Physical Functioning in Central and Eastern Europe: A 10-Year Follow-up of HAPIEE Study — Alcohol Consumption and Longitudinal Trajectories of Physical Functioning in Central and Eastern Europe: A 10-Year Follow-up of HAPIEE Study — Supplementary Data 

# Alcohol Consumption and Longitudinal Trajectories of Physical Functioning in Central and Eastern Europe: A 10-Year Follow-up of HAPIEE Study

## Supplementary Data

Data files

- Supplementary Data - Supplementary Data
