# Supplementary material for: Alcohol Consumption and Longitudinal Trajectories of Physical Functioning in Central and Eastern Europe: A 10-Year Follow-up of HAPIEE Study
Source: J Gerontol A Biol Sci Med Sci. 2016 Jan 8;71(8):1063–8. doi: 10.1093/gerona/glv233 (PMC4945885; doi:10.1093/gerona/glv233)
Supplement: Supplementary Data [file supp_glv233_JGMS_revised_supplementary_tables.docx]

**Supplementary material**

# Supplementary table 1. Sample characteristics in the observed sample

|  | **Country** | | | | | |
| --- | --- | --- | --- | --- | --- | --- |
|  | **Czech Republic** | | **Russia** |  | **Poland** |  |
|  | Men | Women | Men | Women | Men | Women |
| **Total** | 4070 | 4703 | 4239 | 5062 | 5219 | 5490 |
| **Age** (years, %) |  |  |  |  |  |  |
| 45-49 | 15.8 | 17.8 | 15.9 | 18.0 | 17.4 | 19.6 |
| 50-54 | 19.1 | 20.4 | 19.8 | 19.2 | 19.8 | 21.5 |
| 55-59 | 19.8 | 18.5 | 21.6 | 21.6 | 21.5 | 20.6 |
| 60-64 | 22.2 | 23.8 | 19.3 | 18.8 | 20.4 | 19.4 |
| 65-69 | 23.1 | 19.6 | 23.5 | 22.4 | 20.9 | 18.9 |
| Missing | 0 | 0 | 0 | 0 | 0 | 0 |
| **PF-10 score** (mean, SD) |  |  |  |  |  |  |
| Baseline | 85.2 (18.1) | 82.0 (19.3) | 86.9 (18.3) | 77.5 (21.1) | 84.0 (20.2) | 77.0 (21.9) |
| Missing (%) | 1.3 | 1.9 | 0 | 0 | 0.7 | 0.8 |
| Re-examination | 84.8 (15.7) | 81.7 (17.4) | 86.1 (20.0) | 75.8 (22.2) | 77.4 (20.3) | 71.9 (20.9) |
| Missing (%) | 42.9 | 39.8 | 36.3 | 31.9 | 38.8 | 37.8 |
| PQ2009 | 83.2 (20.1) | 80.1 (20.7) | 77.6 (25.9) | 64.6 (26.4) | 76.9 (25.5) | 67.7 (26.5) |
| Missing (%) | 43.4 | 38.5 | 35.4 | 26.8 | 34.8 | 30.8 |
| PQ2012 | 82.6 (20.4) | 80.1 (21.4) | 75.6 (26.4) | 61.8 (27.4) | 73.1 (25.4) | 63.9 (26.6) |
| Missing (%) | 50.4 | 45.1 | 56.9 | 44.9 | 66.9 | 64.4 |
| **Alcohol consumption** |  |  |  |  |  |  |
| Average drinking frequency (%) |  |  |  |  |  |  |
| Never | 6.5 | 18.5 | 13.5 | 17.8 | 22.0 | 46.4 |
| <1/month | 14.1 | 28.0 | 13.9 | 46.0 | 14.5 | 22.7 |
| 1-3/month | 17.1 | 25.5 | 25.7 | 27.9 | 23.5 | 19.0 |
| 1-4/week | 30.5 | 20.7 | 38.5 | 7.9 | 28.6 | 10.2 |
| ≥5/week | 31.9 | 7.2 | 8.5 | 0.5 | 11.4 | 1.7 |
| Annual drinking volume (g, %) |  |  |  |  |  |  |
| 0 | 6.5 | 18.5 | 13.5 | 17.8 | 22.0 | 46.4 |
| 1-1500^a^/1-250^b^ | 31.7 | 29.2 | 28.2 | 31.0 | 39.3 | 25.0 |
| 1501-4000^a^ /251-500^b^ | 17.8 | 12.1 | 19.4 | 28.2 | 18.7 | 10.3 |
| 4001-8000^a^ /501-1500^b^ | 14.8 | 16.2 | 16.2 | 15.0 | 9.6 | 10.2 |
| >8000^a^ />1500^b^ | 29.2 | 24.1 | 22.7 | 8.1 | 10.5 | 8.1 |
| Average drinking quantity/drinking day (%) |  |  |  |  |  |  |
| Non-drinker | 6.5 | 18.5 | 13.5 | 17.8 | 22.0 | 46.4 |
| Light | 66.6 | 33.7 | 24.0 | 19.0 | 58.6 | 30.0 |
| Moderate | 9.5 | 38.0 | 18.2 | 49.4 | 7.0 | 20.3 |
| Heavy | 17.4 | 9.8 | 44.4 | 13.8 | 12.5 | 3.4 |
| Drinking pattern (%) |  |  |  |  |  |  |
| Non-drinker | 6.5 | 18.5 | 13.5 | 17.8 | 22.0 | 46.4 |
| Irregular light-to-moderate | 22.7 | 39.5 | 23.8 | 58.7 | 27.7 | 35.2 |
| Regular light-to-moderate | 28.1 | 12.3 | 17.5 | 4.3 | 22.6 | 7.3 |
| Irregular heavy | 35.0 | 18.9 | 31.3 | 13.0 | 24.3 | 7.7 |
| Regular heavy | 7.7 | 10.8 | 13.9 | 6.2 | 3.4 | 3.4 |
| Missing on alcohol consumption (GF questionnaire, %) | 2.8 | 4.2 | 0 | 0 | 0.6 | 0.5 |
| **Problem drinking** (%) |  |  |  |  |  |  |
| No | 90.8 | 97.9 | 80.8 | 98.6 | 89.9 | 98.7 |
| Yes | 9.2 | 2.1 | 19.2 | 1.4 | 10.1 | 1.3 |
| Missing | 3.9 | 6.2 | 0 | 0 | 13.2 | 27.3 |
| **Education** (%) |  |  |  |  |  |  |
| < Secondary school | 49.8 | 49.3 | 33.2 | 40.1 | 37.0 | 28.6 |
| Secondary school | 31.6 | 40.4 | 34.9 | 33.5 | 32.8 | 44.3 |
| University | 18.0 | 10.0 | 31.9 | 26.4 | 30.1 | 27.0 |
| Missing | 0.6 | 0.5 | 0 | 0 | 0.1 | 0.1 |
| **Number of household amenities** (mean, SD) | 7.1 (2.3) | 6.6 (2.3) | 6.0 (2.2) | 5.4 (2.1) | 6.7 (2.2) | 6.1 (2.2) |
| Missing (%) | 5.9 | 6.3 | 0.8 | 0.3 | 1.4 | 1.7 |
| **Economic activity** (%) |  |  |  |  |  |  |
| Working | 49.6 | 40.4 | 40.3 | 32.4 | 40.8 | 36.1 |
| Pensioner, still working | 9.1 | 7.6 | 21.1 | 16.3 | 7.6 | 5.6 |
| Pensioner, not working | 37.8 | 48.4 | 33.0 | 48.5 | 45.4 | 53.8 |
| Unemployed | 3.2 | 2.6 | 5.6 | 2.8 | 6.0 | 4.3 |
| Missing | 1.3 | 1.0 | 0 | 0 | 0.3 | 0.2 |
| **Self-rated health** (%) |  |  |  |  |  |  |
| Very good/good | 39.4 | 40.4 | 15.8 | 5.8 | 40.0 | 31.7 |
| Average | 48.3 | 48.1 | 67.3 | 67.2 | 46.0 | 52.2 |
| Poor/very poor | 11.8 | 10.8 | 16.9 | 27.0 | 13.9 | 15.9 |
| Missing | 0.5 | 0.7 | 0 | 0 | 0.2 | 0.2 |
| **Long-standing illness** (%) |  |  |  |  |  |  |
| No | 42.9 | 37.3 | 65.1 | 51.8 | 45.9 | 36.6 |
| Yes | 55.8 | 61.3 | 34.9 | 48.2 | 53.5 | 62.8 |
| Missing | 1.3 | 1.3 | 0 | 0 | 0.7 | 0.6 |
| **Spine/joint problems** (%) |  |  |  |  |  |  |
| No | 47.6 | 38.7 | 40.9 | 29.3 | 36.7 | 24.5 |
| Yes | 50.0 | 58.6 | 59.1 | 70.7 | 62.8 | 75.3 |
| Missing | 2.4 | 2.7 | 0 | 0 | 0.4 | 0.3 |
| **Cardiovascular disease** (%) |  |  |  |  |  |  |
| No | 80.3 | 84.5 | 76.4 | 79.9 | 75.3 | 77.9 |
| Yes | 16.4 | 10.0 | 23.6 | 20.1 | 23.8 | 21.2 |
| Missing | 3.3 | 5.5 | 0 | 0 | 0.9 | 0.9 |
| **Hypertension** (%) |  |  |  |  |  |  |
| No | 21.9 | 23.6 | 36.6 | 33.3 | 29.1 | 38.3 |
| Yes | 58.5 | 48.6 | 63.2 | 66.7 | 57.0 | 48.0 |
| Missing | 19.6 | 16.9 | 0.2 | 0.1 | 14.0 | 13.7 |
| **Cancer** (%) |  |  |  |  |  |  |
| No | 91.8 | 86.6 | 98.7 | 96.0 | 95.8 | 93.1 |
| Yes | 3.9 | 7.7 | 1.3 | 4.0 | 3.2 | 6.1 |
| Missing | 4.3 | 5.6 | 0 | 0 | 1.0 | 0.8 |

^a^ among men; ^b^ among women

# Supplementary table 2. Age-adjusted associations between alcohol consumption and physical functioning trajectories

|  | **Men (coefficient, SE)** | | | **Women (coefficient, SE)** | | |
| --- | --- | --- | --- | --- | --- | --- |
|  | **Czech Republic** | **Russia** | **Poland** | **Czech Republic** | **Russia** | **Poland** |
| **Initial status**^§^ | 92.25 (0.66)^***^ | 94.30 (0.73)^***^ | 90.86 (0.65)^***^ | 89.86 (0.81)^***^ | 89.03 (1.20)^***^ | 88.91 (0.95)^***^ |
| **Slope**^§^ | -0.35 (0.10)^**^ | -0.91 (0.17)^***^ | -1.02 (0.12)^***^ | -0.18 (0.12) | -1.38 (0.24)^***^ | -1.07 (0.18)^***^ |
| **Average drinking quantity/drinking day**  (reference group: light drinker) |  |  |  |  |  |  |
| ***Initial status*** |  |  |  |  |  |  |
| Non-drinker | -9.62 (1.54)^***^ | -3.56 (1.11)^**^ | -5.20 (0.79)^***^ | -7.48 (0.94)^***^ | -7.21 (1.03)^***^ | -3.90 (0.66)^***^ |
| Moderate | 0.43 (0.81) | 3.67 (0.79)^***^ | 1.71 (0.89) | 0.21 (0.57) | 1.38 (0.74) | 2.52 (0.68)^***^ |
| Heavy | -0.75 (0.70) | 2.24 (0.70)^**^ | 0.76 (0.74) | -0.27 (0.83) | 1.76 (0.95) | 1.07 (1.24) |
| ***Slope*** |  |  |  |  |  |  |
| Non-drinker | -0.08 (0.21) | -0.61 (0.20)^**^ | -0.10 (0.13) | -0.14 (0.13) | -0.16 (0.18) | -0.27 (0.11)^*^ |
| Moderate | -0.32 (0.14)^*^ | -0.34 (0.17)^*^ | -0.27 (0.17) | -0.02 (0.08) | -0.16 (0.14) | -0.28 (0.12)^*^ |
| Heavy | -0.12 (0.11) | -0.49 (0.14)^**^ | -0.28 (0.14)^*^ | -0.10 (0.13) | -0.22 (0.18) | -0.41 (0.23) |
| **Drinking pattern**  (reference group: regular light-to-moderate drinker) |  |  |  |  |  |  |
| ***Initial status*** |  |  |  |  |  |  |
| Non-drinker | -9.68 (1.59)^***^ | -6.24 (1.10)^***^ | -5.66 (0.88)^***^ | -8.34 (1.08)^***^ | -11.65 (1.38)^***^ | -7.22 (0.99)^***^ |
| Irregular light-to-moderate | -1.85 (0.79)^*^ | -3.31 (0.85)^***^ | -1.46 (0.70)^*^ | -1.91 (0.80)^*^ | -3.94 (1.15)^**^ | -3.07 (0.95)^**^ |
| Irregular heavy | 0.90 (0.64) | 0.39 (0.72) | 1.32 (0.67) | 1.21 (0.83) | -1.65 (1.29) | -2.02 (1.17) |
| Regular heavy | -0.30 (1.02) | 0.02 (0.84) | 0.14 (1.38) | -0.04 (0.94) | -2.74 (1.47) | 0.09 (1.49) |
| ***Slope*** |  |  |  |  |  |  |
| Non-drinker | -0.16 (0.21) | -0.36 (0.21) | -0.17 (0.15) | -0.26 (0.15) | 0.17 (0.27) | -0.18 (0.18) |
| Irregular light-to-moderate | -0.17 (0.12) | 0.14 (0.18) | -0.02 (0.12) | -0.14 (0.11) | 0.24 (0.23) | 0.01 (0.17) |
| Irregular heavy | -0.16 (0.10) | -0.14 (0.17) | -0.31 (0.14)^*^ | -0.14 (0.13) | 0.18 (0.26) | -0.18 (0.22) |
| Regular heavy | -0.39 (0.18)^*^ | -0.36 (0.22) | -0.80 (0.30)^**^ | -0.21 (0.15) | -0.06 (0.30) | -0.20 (0.28) |
| **Problem drinking**^a^  (reference group: non-problem drinking) |  |  |  |  |  |  |
| ***Initial status*** |  |  |  |  |  |  |
| Problem drinking | -1.69 (0.90) | 0.56 (0.63) | -1.89 (0.94)^*^ |  |  |  |
| ***Slope*** |  |  |  |  |  |  |
| Problem drinking | -0.11 (0.14) | -0.25 (0.16) | -0.32 (0.21) |  |  |  |
| **Past drinking behavior**  (reference group: continuing drinker) |  |  |  |  |  |  |
| ***Intercept*** |  |  |  |  |  |  |
| Lifetime abstainer |  | -6.26 (3.41) |  |  | -5.89 (1.19)^***^ |  |
| Former drinker, health reasons |  | -15.19 (1.77)^***^ |  |  | -15.90 (1.56)^***^ |  |
| Former drinker, non-health reasons |  | -1.32 (0.99) |  |  | -7.25 (1.60)^***^ |  |
| Reduced drinker, health reasons |  | -10.21 (0.92)^***^ |  |  | -7.18 (0.98)^***^ |  |
| Reduced drinker, non-health reasons |  | -0.57 (0.59) |  |  | 0.85 (0.72) |  |
| ***Slope*** |  |  |  |  |  |  |
| Lifetime abstainer |  | 0.27 (0.58) |  |  | -0.03 (0.21) |  |
| Former drinker, health reasons |  | -0.02 (0.29) |  |  | 0.03 (0.25) |  |
| Former drinker, non-health reasons |  | -0.48 (0.22)^*^ |  |  | 0.24 (0.27) |  |
| Reduced drinker, health reasons |  | 0.10 (0.19) |  |  | 0.34 (0.18)^*^ |  |
| Reduced drinker, non-health reasons |  | 0.16 (0.13) |  |  | 0.20 (0.14) |  |

^*^ p<0.05, ^**^ p<0.01, ^***^ p<0.001; ^§^conditional on age; ^a^ among male drinkers; SE: standard error

# Supplementary table 3. Multivariable-adjusted associations of average drinking frequency and annual drinking volume with physical functioning trajectories

|  | **Men (coefficient, SE)** | | | **Women (coefficient, SE)** | | |
| --- | --- | --- | --- | --- | --- | --- |
|  | **Czech Republic** | **Russia** | **Poland** | **Czech Republic** | **Russia** | **Poland** |
| **Average drinking frequency**  (reference group: 1-3/month) |  |  |  |  |  |  |
| ***Initial status*** |  |  |  |  |  |  |
| 0 | -5.76 (1.47)^***^ | -4.06 (0.99)^***^ | -4.40 (0.81)^***^ | -4.87 (0.83)^***^ | -7.28 (0.89)^***^ | -4.74 (0.69)^***^ |
| <1/month | -0.65 (0.87) | -2.29 (0.89)^*^ | -1.67 (0.78)^*^ | -1.27 (0.59)^*^ | -0.76 (0.58) | -2.50 (0.70)^***^ |
| 1-4/week^a^ | 0.46 (0.69) | 0.57 (0.60) | -0.35 (0.61) |  |  |  |
| ≥5/week^a^ | 1.40 (0.66)^*^ | 0.61 (0.88) | -0.40 (0.76) |  |  |  |
| ≥1/week^b^ |  |  |  | -0.19 (0.56) | 0.77 (0.90) | 0.12 (0.80) |
| ***Slope*** |  |  |  |  |  |  |
| 0 | 0.14 (0.23) | -0.21 (0.20) | 0.05 (0.14) | -0.04 (0.13) | -0.09 (0.17) | 0.07 (0.14) |
| <1/month | 0.16 (0.15) | 0.11 (0.18) | 0.15 (0.14) | 0.06 (0.10) | -0.16 (0.12) | 0.16 (0.13) |
| 1-4/week^a^ | 0.19 (0.12) | 0.03 (0.14) | 0.05 (0.11) |  |  |  |
| ≥5/week^a^ | 0.14 (0.12) | -0.09 (0.21) | -0.12 (0.15) |  |  |  |
| ≥1/week^b^ |  |  |  | 0.09 (0.09) | -0.37 (0.19)^*^ | -0.05 (0.15) |
| **Annual drinking volume (g)**  (reference group: 1-1500^a^/1-250^b^) |  |  |  |  |  |  |
| ***Initial status*** |  |  |  |  |  |  |
| 0 | -5.56 (1.41)^***^ | -2.18 (1.00)^*^ | -3.24 (0.75)^***^ | -3.45 (0.82)^***^ | -5.29 (0.90)^***^ | -2.16 (0.65)^**^ |
| 1501-4000^a^ /251-500^b^ | 0.39 (0.69) | 1.96 (0.74)^**^ | 1.47 (0.60)^*^ | 1.46 (0.77) | 2.81 (0.65)^***^ | 2.86 (0.80)^***^ |
| 4001-8000^a^ /501-1500^b^ | 1.35 (0.70) | 3.53 (0.74)^***^ | 1.62 (0.76)^*^ | 1.17 (0.65) | 3.11 (0.79)^***^ | 3.00 (0.81)^***^ |
| >8000^a^ />1500^b^ | 1.08 (0.58) | 2.68 (0.69)^***^ | 0.75 (0.77) | 1.12 (0.60) | 1.71 (0.99) | 2.62 (0.91)^**^ |
| ***Slope*** |  |  |  |  |  |  |
| 0 | 0.04 (0.22) | -0.27 (0.20) | -0.05 (0.13) | -0.10 (0.14) | -0.02 (0.16) | -0.09 (0.12) |
| 1501-4000^a^ /251-500^b^ | 0.05 (0.12) | 0.05 (0.16) | -0.11 (0.11) | -0.05 (0.12) | -0.09 (0.13) | -0.13 (0.15) |
| 4001-8000^a^ /501-1500^b^ | 0.03 (0.12) | -0.10 (0.17) | -0.15 (0.15) | -0.03 (0.11) | -0.04 (0.16) | -0.23 (0.16) |
| >8000^a^ />1500^b^ | 0.07 (0.10) | -0.11 (0.16) | -0.26 (0.15) | 0.01 (0.10) | -0.24 (0.20) | -0.23 (0.17) |

^*^ p<0.05, ^**^ p<0.01, ^***^ p<0.001, ^§^ conditional on covariates; ^a^ among men; ^b^ among women; SE: standard error

Adjusted for age, educational attainment, household amenities, economic activity, marital status, spine/joint problems, BMI and smoking status

# Supplementary table 4. Multivariable-adjusted associations between past drinking behavior combined with drinking pattern and physical functioning trajectories in the Russian cohort

|  | **Coefficient (SE)** | |
| --- | --- | --- |
|  | **Men** | **Women** |
| **Past drinking behavior**  (reference group: regular light-to-moderate drinker) |  |  |
| ***Initial status*** |  |  |
| Mean^§^ | 93.15 (1.43)^***^ | 88.75 (1.61)^***^ |
| Lifetime abstainer | -5.51 (2.95) | -4.84 (1.50)^**^ |
| Former drinker, health reasons | -12.18 (1.69)^***^ | -12.76 (1.80)^***^ |
| Former drinker, non-health reasons | -1.33 (1.11) | -6.28 (1.81)^**^ |
| Reduced drinker, health reasons | -8.49 (1.03)^***^ | -5.54 (1.36)^***^ |
| Reduced drinker, non-health reasons | -0.46 (0.83) | 1.03 (1.21) |
| Continuing drinker |  |  |
| Irregular light-to-moderate | -2.23 (1.02)^*^ | -0.72 (1.13) |
| Irregular heavy | -1.01 (0.95) | 2.39 (1.58) |
| Regular heavy | -0.09 (0.84) | 2.15 (1.26) |
| ***Slope*** |  |  |
| Mean^§^ | -0.88 (0.35)^*^ | -1.65 (0.33)^***^ |
| Lifetime abstainer | 0.32 (0.58) | 0.18 (0.30) |
| Former drinker, health reasons | 0.21 (0.35) | 0.26 (0.34) |
| Former drinker, non-health reasons | -0.15 (0.27) | 0.41 (0.35) |
| Reduced drinker, health reasons | 0.30 (0.26) | 0.45 (0.38) |
| Reduced drinker, non-health reasons | 0.37 (0.22) | 0.30 (0.25) |
| Continuing drinker |  |  |
| Irregular light-to-moderate | 0.32 (0.25) | 0.19 (0.25) |
| Irregular heavy | 0.32 (0.25) | -0.06 (0.33) |
| Regular heavy | 0.21 (0.23) | 0.19 (0.28) |

^*^ p<0.05, ^**^ p<0.01, ^***^ p<0.001; ^§^ conditional on covariates; SE: standard error

Adjusted for age, educational attainment, economic activity, household amenities, marital status,

spine/joint problems, BMI and smoking status.

# Supplementary table 5. Multivariable-adjusted associations between alcohol consumption and physical functioning trajectories, additionally controlled for selected baseline health conditions

|  | **Men (coefficient, SE)** | | | **Women (coefficient, SE)** | | |
| --- | --- | --- | --- | --- | --- | --- |
|  | **Czech Republic** | **Russia** | **Poland** | **Czech Republic** | **Russia** | **Poland** |
| **Initial status**^§^ | 97.74 (1.10)^***^ | 97.18 (1.37)^***^ | 93.79 (1.17)^***^ | 94.28 (1.20)^***^ | 97.84 (1.82)^***^ | 98.11 (1.45)^***^ |
| **Slope**^§^ | -0.20 (0.22) | -0.71 (0.36)^*^ | -1.27 (0.27)^***^ | -0.19 (0.23) | -1.80 (0.39)^***^ | -1.65 (0.31)^***^ |
| **Average drinking frequency**  (reference group: 1-3/month) |  |  |  |  |  |  |
| ***Initial status*** |  |  |  |  |  |  |
| 0 | -3.02 (1.23)^*^ | -2.12 (0.80)^**^ | -1.76 (0.70)^*^ | -2.83 (0.72)^***^ | -3.18 (0.79)^***^ | -2.16 (0.63)^***^ |
| <1/month | -0.47 (0.75) | -1.00 (0.76) | -0.54 (0.69) | -0.66 (0.52) | -0.30 (0.56) | -1.50 (0.64)^*^ |
| 1-4/week^a^ | 0.14 (0.60) | 0.10 (0.56) | -0.15 (0.54) |  |  |  |
| ≥5/week^a^ | 0.59 (0.57) | -0.38 (0.85) | -0.47 (0.68) |  |  |  |
| ≥1/week^b^ |  |  |  | -0.15 (0.49) | 0.58 (0.89) | 0.70 (0.73) |
| ***Slope*** |  |  |  |  |  |  |
| 0 | 0.11 (0.22) | -0.26 (0.20) | -0.02 (0.14) | -0.06 (0.13) | -0.23 (0.17) | 0.00 (0.14) |
| <1/month | 0.14 (0.15) | 0.10 (0.18) | 0.14 (0.14) | 0.06 (0.10) | -0.18 (0.12) | 0.14 (0.13) |
| 1-4/week^a^ | 0.17 (0.12) | 0.03 (0.14) | 0.05 (0.11) |  |  |  |
| ≥5/week^a^ | 0.12 (0.12) | -0.08 (0.21) | -0.10 (0.15) |  |  |  |
| ≥1/week^b^ |  |  |  | 0.09 (0.09) | -0.36 (0.19) | -0.07 (0.15) |
| **Annual drinking volume (g)**  (reference group: 1-1500^a^/1-250^b^) |  |  |  |  |  |  |
| ***Initial status*** |  |  |  |  |  |  |
| 0 | -3.02 (1.17)^*^ | -1.27 (0.87) | -1.30 (0.65)^*^ | -2.02 (0.71)^**^ | -2.74 (0.80)^**^ | -0.44 (0.60) |
| 1501-4000^a^ /251-500^b^ | -0.15 (0.61) | 0.82 (0.68) | 0.76 (0.53) | 0.99 (0.67) | 1.65 (0.61)^**^ | 2.12 (0.74)^**^ |
| 4001-8000^a^ /501-1500^b^ | 0.46 (0.60) | 1.77 (0.76)^*^ | 0.28 (0.70) | 0.78 (0.57) | 0.69 (0.74) | 2.45 (0.75)^**^ |
| >8000^a^ />1500^b^ | 0.14 (0.50) | 0.70 (0.66) | 0.10 (0.70) | 0.53 (0.52) | 0.28 (0.92) | 2.37 (0.82)^**^ |
| ***Slope*** |  |  |  |  |  |  |
| 0 | 0.02 (0.21) | -0.31 (0.20) | -0.11 (0.13) | -0.11 (0.14) | -0.12 (0.16) | -0.14 (0.12) |
| 1501-4000^a^ /251-500^b^ | 0.04 (0.11) | 0.04 (0.16) | -0.09 (0.11) | -0.05 (0.12) | -0.07 (0.13) | -0.12 (0.15) |
| 4001-8000^a^ /501-1500^b^ | 0.04 (0.12) | -0.09 (0.17) | -0.11 (0.15) | -0.03 (0.11) | 0.03 (0.16) | -0.22 (0.16) |
| >8000^a^ />1500^b^ | 0.06 (0.10) | -0.10 (0.16) | -0.24 (0.15) | 0.02 (0.10) | -0.20 (0.20) | -0.23 (0.17) |
| **Average drinking quantity/drinking day**  (reference group: light drinker) |  |  |  |  |  |  |
| ***Initial status*** |  |  |  |  |  |  |
| Non-drinker | -3.11 (1.15)^**^ | -0.88 (0.83) | -1.27 (0.62)^*^ | -2.42 (0.69)^***^ | -2.57 (0.85)^**^ | -0.63 (0.58) |
| Moderate | 0.77 (0.64) | 1.23 (0.68) | 1.43 (0.75) | 0.14 (0.44) | 1.14 (0.63) | 2.16 (0.59)^***^ |
| Heavy | -0.45 (0.56) | 1.61 (0.58)^**^ | 0.90 (0.63) | 0.18 (0.69) | 1.08 (0.85) | 2.90 (1.04)^**^ |
| ***Slope*** |  |  |  |  |  |  |
| Non-drinker | -0.04 (0.20) | -0.45 (0.20)^*^ | -0.09 (0.13) | -0.10 (0.13) | -0.14 (0.18) | -0.15 (0.12) |
| Moderate | -0.25 (0.14) | -0.24 (0.17) | -0.18 (0.17) | 0.01 (0.08) | -0.10 (0.14) | -0.24 (0.12)^*^ |
| Heavy | -0.03 (0.11) | -0.24 (0.14) | -0.16 (0.14) | 0.00 (0.13) | -0.01 (0.18) | -0.30 (0.23) |
| **Drinking pattern**  (reference group: regular light-to-moderate drinker) |  |  |  |  |  |  |
| ***Initial status*** |  |  |  |  |  |  |
| Non-drinker | -3.36 (1.19)^**^ | -2.22 (0.85)^**^ | -1.09 (0.70) | -2.77 (0.83)^**^ | -3.87 (1.27)^**^ | -3.36 (0.86)^***^ |
| Irregular light-to-moderate | -0.72 (0.60) | -1.31 (0.70) | 0.07 (0.58) | -0.66 (0.63) | -0.60 (1.11) | -2.21 (0.83)^**^ |
| Irregular heavy | -0.11 (0.51) | 0.39 (0.64) | 1.21 (0.57)^*^ | 0.54 (0.67) | 0.55 (1.23) | -1.86 (1.01) |
| Regular heavy | -0.69 (0.82) | -0.19 (0.78) | 2.07 (1.25) | -0.25 (0.75) | -1.15 (1.40) | 0.09 (1.26) |
| ***Slope*** |  |  |  |  |  |  |
| Non-drinker | -0.12 (0.21) | -0.28 (0.21) | -0.14 (0.15) | -0.21 (0.15) | 0.23 (0.27) | 0.03 (0.18) |
| Irregular light-to-moderate | -0.16 (0.12) | 0.09 (0.18) | -0.02 (0.12) | -0.12 (0.11) | 0.33 (0.24) | 0.13 (0.17) |
| Irregular heavy | -0.10 (0.10) | 0.00 (0.17) | -0.21 (0.14) | -0.11 (0.13) | 0.40 (0.27) | -0.05 (0.22) |
| Regular heavy | -0.27 (0.18) | -0.16 (0.22) | -0.63 (0.30)^*^ | -0.13 (0.15) | 0.14 (0.31) | -0.13 (0.28) |
| **Problem drinking**^c^  (reference group: non-problem drinking) |  |  |  |  |  |  |
| ***Initial status*** |  |  |  |  |  |  |
| Problem drinking | -0.26 (0.69) | 0.67 (0.67) | -0.48 (0.80) |  |  |  |
| ***Slope*** |  |  |  |  |  |  |
| Problem drinking | -0.09 (0.14) | -0.11 (0.16) | -0.20 (0.21) |  |  |  |
| **Past drinking behavior**  (reference group: continuing drinker) |  |  |  |  |  |  |
| ***Intercept*** |  |  |  |  |  |  |
| Lifetime abstainer |  | -2.37 (2.57) |  |  | -1.59 (0.96) |  |
| Former drinker, health reasons |  | -6.01 (1.29)^***^ |  |  | -7.50 (1.26)^***^ |  |
| Former drinker, non-health reasons |  | 0.26 (0.82) |  |  | -3.07 (1.29)^*^ |  |
| Reduced drinker, health reasons |  | -3.15 (0.77)^***^ |  |  | -2.28 (0.85)^**^ |  |
| Reduced drinker, non-health reasons |  | 0.59 (0.52) |  |  | 0.83 (0.65) |  |
| ***Slope*** |  |  |  |  |  |  |
| Lifetime abstainer |  | 0.01 (0.54) |  |  | -0.09 (0.21) |  |
| Former drinker, health reasons |  | -0.06 (0.29) |  |  | -0.05 (0.25) |  |
| Former drinker, non-health reasons |  | -0.39 (0.22) |  |  | 0.12 (0.27) |  |
| Reduced drinker, health reasons |  | 0.07 (0.19) |  |  | 0.21 (0.17) |  |
| Reduced drinker, non-health reasons |  | 0.14 (0.14) |  |  | 0.14 (0.14) |  |

^*^ p<0.05, ^**^ p<0.01, ^***^ p<0.001, ^§^ conditional on covariates; ^a^ among men; ^b^ among women; ^c^ among male drinkers; SE: standard error

Adjusted for age, educational attainment, household amenities, economic activity, marital status, self-rated health, spine/joint problems, prevalence of CVD, history of cancer, BMI and smoking status

# Supplementary table 6. GF-based alcohol indices, problem drinking and serum gamma-glutamyl transferase at baseline

|  | **Men** | | |  | | | **Women** | | |  | | |
| --- | --- | --- | --- | --- | --- | --- | --- | --- | --- | --- | --- | --- |
|  | **GGT (Russia)** | | | **GGT (Czech Republic & Poland)** | | | **GGT (Russia)** | | | **GGT (Czech Republic & Poland)** | | |
|  | Mean | SD | N | Mean | SD | N | Mean | SD | N | Mean | SD | N |
| **Average drinking frequency** |  |  |  |  |  |  |  |  |  |  |  |  |
| Never | 30.07 | 35.02 | 569 | 31.64 | 65.31 | 471 | 27.32 | 21.31 | 892 | 24.40 | 54.84 | 532 |
| <1/month | 32.34 | 27.22 | 585 | 28.79 | 48.54 | 452 | 29.29 | 30.42 | 2313 | 23.24 | 44.49 | 377 |
| 1-3/month | 37.49 | 47.36 | 1086 | 35.31 | 57.33 | 597 | 28.90 | 23.29 | 1407 | 18.90 | 42.88 | 308 |
| 1-4/week | 43.08 | 46.98 | 1621 | 42.32 | 74.83 | 809 | 33.01 | 32.91 | 397 | 34.44 | 91.38 | 203 |
| ≥5/week | 55.13 | 83.58 | 359 | 57.98 | 100.23 | 619 | 33.00 | 22.83 | 24 | 64.90 | 156.97 | 49 |
| **Annual drinking volume** (g) |  |  |  |  |  |  |  |  |  |  |  |  |
| 0 | 30.07 | 35.02 | 569 | 31.64 | 65.31 | 471 | 27.32 | 21.31 | 892 | 24.40 | 54.84 | 532 |
| 1-1500 | 33.02 | 31.88 | 1188 | 31.95 | 57.07 | 1099 | 28.13 | 25.48 | 1559 | 22.62 | 47.92 | 419 |
| 1501-4000 | 39.94 | 52.05 | 820 | 42.51 | 75.83 | 498 | 29.21 | 30.71 | 1417 | 18.83 | 33.51 | 161 |
| 4001-8000 | 42.55 | 46.19 | 683 | 40.33 | 67.51 | 335 | 30.46 | 26.10 | 759 | 18.83 | 35.67 | 167 |
| >8000 | 50.21 | 64.89 | 960 | 63.17 | 102.40 | 545 | 34.32 | 34.13 | 406 | 47.90 | 122.19 | 190 |
| **Average drinking quantity/drinking day** |  |  |  |  |  |  |  |  |  |  |  |  |
| Non-drinker | 30.07 | 35.02 | 569 | 31.64 | 65.31 | 471 | 27.32 | 21.31 | 892 | 24.40 | 54.84 | 532 |
| Light | 36.46 | 42.84 | 1011 | 39.07 | 74.41 | 1864 | 28.15 | 22.85 | 958 | 23.19 | 58.10 | 455 |
| Moderate | 40.77 | 53.47 | 765 | 47.00 | 59.23 | 209 | 28.86 | 26.12 | 2486 | 26.63 | 67.96 | 402 |
| Heavy | 43.31 | 51.70 | 1875 | 53.41 | 84.82 | 404 | 33.86 | 40.42 | 697 | 43.72 | 105.04 | 80 |
| **Drinking pattern** |  |  |  |  |  |  |  |  |  |  |  |  |
| Non-drinker | 30.07 | 35.02 | 569 | 31.64 | 65.31 | 471 | 27.32 | 21.31 | 892 | 24.40 | 54.84 | 532 |
| Irregular light-to-moderate | 31.37 | 25.63 | 546 | 28.87 | 50.33 | 452 | 28.57 | 25.96 | 2366 | 22.96 | 42.71 | 384 |
| Regular light-to-moderate | 40.23 | 50.60 | 1195 | 40.79 | 71.53 | 1150 | 28.29 | 23.09 | 806 | 23.40 | 63.37 | 311 |
| Irregular heavy | 36.48 | 27.97 | 645 | 40.15 | 76.38 | 552 | 31.97 | 37.90 | 660 | 25.27 | 67.86 | 161 |
| Regular heavy | 47.84 | 63.12 | 1265 | 68.44 | 103.89 | 323 | 34.95 | 34.32 | 309 | 56.73 | 137.95 | 81 |
| **Problem drinking** |  |  |  |  |  |  |  |  |  |  |  |  |
| No | 36.69 | 39.75 | 3409 | 37.04 | 65.71 | 2503 | 28.89 | 26.52 | 4963 | 25.86 | 63.30 | 1230 |
| Yes | 50.93 | 72.98 | 811 | 84.72 | 134.23 | 230 | 46.96 | 59.56 | 70 | 90.65 | 190.72 | 20 |

GGT: gamma-glutamyl transferase (IU/L); SD: standard deviation; N: sample size

Please note that the GGT in Russia was analyzed in a different laboratory from the GGT in Czech Republic and Poland.

# Supplementary table 7. PF-10 score and objective physical performances at re-examination

|  | **Grip strength (unit)** | | | **5 chair stands (seconds)** | | |
| --- | --- | --- | --- | --- | --- | --- |
|  | Mean | SD | N | Mean | SD | N |
| **Quartiles of PF-10 score at re-examination**^*^ |  |  |  |  |  |  |
| **Czech Republic** |  |  |  |  |  |  |
| 1^st^ | 28.77 | 10.59 | 1251 | 12.54 | 4.57 | 1005 |
| 2^nd^ | 31.94 | 9.93 | 1266 | 10.37 | 4.45 | 1247 |
| 3^rd^ | 34.05 | 10.09 | 1280 | 9.44 | 3.12 | 1265 |
| 4^th^ | 37.33 | 10.87 | 1281 | 8.53 | 2.59 | 1270 |
| **Russia** |  |  |  |  |  |  |
| 1^st^ | 27.63 | 9.35 | 1604 | 13.28 | 4.08 | 1240 |
| 2^nd^ | 32.63 | 9.84 | 1985 | 11.48 | 3.21 | 1899 |
| 3^rd^ | 37.47 | 10.40 | 1005 | 10.55 | 2.77 | 994 |
| 4^th^ | 39.93 | 10.73 | 1402 | 10.24 | 2.84 | 1385 |
| **Poland** |  |  |  |  |  |  |
| 1^st^ | 26.93 | 10.24 | 1564 | 13.46 | 5.24 | 1265 |
| 2^nd^ | 30.90 | 9.95 | 1611 | 11.03 | 3.55 | 1550 |
| 3^rd^ | 34.84 | 10.10 | 1613 | 10.10 | 3.04 | 1579 |
| 4^th^ | 36.72 | 10.73 | 1600 | 9.51 | 2.89 | 1577 |

^*^ Country-specific quartiles; SD: standard deviation; N: sample size
